# deep-Sep: a deep learning-based method for fast and accurate prediction of selenoprotein genes in bacteria

**DOI:** 10.1128/msystems.01258-24

**Published:** 2025-03-10

**Authors:** Yao Xiao, Yan Zhang

**Affiliations:** 1 Shenzhen Key Laboratory of Marine Bioresources and Ecology, Brain Disease and Big Data Research Institute, College of Life Sciences and Oceanography, Shenzhen University, Shenzhen, Guangdong, China; 2 Shenzhen-Hong Kong Institute of Brain Science-Shenzhen Fundamental Research Institutions, Shenzhen, Guangdong, China; Institut de Recherche pour le Developpement Delegation Regionale Occitanie, Montpellier, France

**Keywords:** selenium, selenoprotein, UGA codon, deep learning, bacteria

## Abstract

Selenoproteins are a special group of proteins with major roles in cellular antioxidant defense. They contain the 21st amino acid selenocysteine (Sec) in the active sites, which is encoded by an in-frame UGA codon. Compared to eukaryotes, identification of selenoprotein genes in bacteria remains challenging due to the absence of an effective strategy for distinguishing the Sec-encoding UGA codon from a normal stop signal. In this study, we have developed a deep learning-based algorithm, deep-Sep, for quickly and precisely identifying selenoprotein genes in bacterial genomic sequences. This algorithm uses a Transformer-based neural network architecture to construct an optimal model for detecting Sec-encoding UGA codons and a homology search-based strategy to remove additional false positives. During the training and testing stages, deep-Sep has demonstrated commendable performance, including an *F*_1_ score of 0.939 and an area under the receiver operating characteristic curve of 0.987. Furthermore, when applied to 20 bacterial genomes as independent test data sets, deep-Sep exhibited remarkable capability in identifying both known and new selenoprotein genes, which significantly outperforms the existing state-of-the-art method. Our algorithm has proved to be a powerful tool for comprehensively characterizing selenoprotein genes in bacterial genomes, which should not only assist in accurate annotation of selenoprotein genes in genome sequencing projects but also provide new insights for a deeper understanding of the roles of selenium in bacteria.

**IMPORTANCE:**

Selenium is an essential micronutrient present in selenoproteins in the form of Sec, which is a rare amino acid encoded by the opal stop codon UGA. Identification of all selenoproteins is of vital importance for investigating the functions of selenium in nature. Previous strategies for predicting selenoprotein genes mainly relied on the identification of a special *cis*-acting Sec insertion sequence (SECIS) element within mRNAs. However, due to the complexity and variability of SECIS elements, recognition of all selenoprotein genes in bacteria is still a major challenge in the annotation of bacterial genomes. We have developed a deep learning-based algorithm to predict selenoprotein genes in bacterial genomic sequences, which demonstrates superior performance compared to currently available methods. This algorithm can be utilized in either web-based or local (standalone) modes, serving as a promising tool for identifying the complete set of selenoprotein genes in bacteria.

## INTRODUCTION

Selenocysteine (Sec) is the 21st naturally occurring amino acid, which exists in all three kingdoms of life. It has been identified as the major biological form of the trace element selenium (Se) in several enzymes and proteins found in a wide range of organisms from bacteria to humans (1, 2). These proteins, known as selenoproteins, play critical roles in many biological processes, such as redox homeostasis and signaling, immune responses, inflammation, and hormone metabolism (3–5). The biosynthesis of Sec and its co-translational insertion into proteins involve a complex molecular machinery that recodes in-frame UGA codons (normally function as stop signals) to serve as Sec codons (6–10). In bacteria, this process requires a Sec insertion sequence (SECIS) element (a *cis*-acting stem-loop structure located immediately downstream of Sec-encoding UGA codon) and several *trans*-acting factors including Sec synthase (SelA), Sec-specific elongation factor (SelB), tRNA^[Ser]Sec^, and selenophosphate synthetase (SelD). In archaea and eukaryotes, SECIS elements are located in the 3'-untranslated regions (UTRs) of selenoprotein genes. In addition, somewhat different steps and enzymes, such as archaeal/eukaryotic Sec synthase (SecS), archaeal/eukaryotic Sec-specific elongation factor (EFSec), and *O*-phosphoseryl-tRNA^Sec^ kinase (PSTK), are needed for the incorporation of Sec into selenoproteins.

In the past two decades, more than 100 selenoprotein families (mostly in bacteria) have been reported in various prokaryotic and eukaryotic organisms, many of which were identified using bioinformatics approaches (11–15). Both SECIS-dependent and SECIS-independent algorithms have been developed to predict selenoprotein genes in genomic databases (16, 17). The general principle of the SECIS-based approach is to recognize potential SECIS elements with predefined sequence and structural features, then to examine genomic context to identify appropriate protein-coding regions, and finally to select good candidates for selenoprotein genes. Based on highly conserved features of eukaryotic SECIS elements, a reliable tool named SECISearch and its successor SECISearch3 were developed for the prediction of eukaryotic SECIS elements and selenoprotein genes (11, 18). Similarly, a SECIS-based program (named archaeal SECISearch) was also developed for archaeal selenoprotein gene prediction (14). In contrast, identification of common features in bacterial SECIS elements has proved to be very difficult as some of them bear no resemblance to each other (19–21). Although a consensus structural model for bacterial SECIS elements and a related tool named bSECISearch (currently the only available and state-of-the-art method for predicting bacterial selenoprotein genes) have been previously proposed, a small number of selenoprotein genes contain SECIS elements that do not meet the constraints of this model, leading to inaccuracies in their recognition (13). The SECIS-independent approach adopts a homology-based strategy to search for cysteine (Cys)/TGA pairs in nucleotide sequence data sets by utilizing a vast collection of Cys-containing proteins, which is based on the fact that almost all selenoproteins have numerous homologs in which Sec is replaced with Cys (14, 22, 23). Additional criteria are used to remove false positives and to discover new selenoprotein genes. Although a large number of selenoprotein genes have been identified in both completely sequenced bacterial genomes and environmental metagenomes via SECIS-dependent and SECIS-independent approaches, both strategies have significant drawbacks: (i) they are very time-consuming and require significant computational time and resources, especially the SECIS-independent approach which can take several days or even weeks to complete; (ii) they often suffer from a relatively high rate of false positives, which leads to large uncertainty in the selection of new selenoprotein candidates; (iii) they are not capable of identifying all selenoprotein genes, e.g., selenoprotein genes with atypical SECIS elements are frequently overlooked by SECIS-dependent algorithms. Therefore, these existing methods are not well suited for efficient selenoprotein gene prediction and annotation in bacterial genome sequencing projects. With the exponential increase in the volume of genomic data, it becomes more and more urgent to develop a fast, accurate, and definitive algorithm for the identification of the complete set of selenoprotein genes (known as selenogenome) in sequenced organisms, particularly bacteria.

In recent years, deep learning has made remarkable progress in a wide range of biological fields. Sequence- and structure-based deep learning methods have been used in trace element research for a variety of applications such as metalloprotein prediction, metal-binding RNA prediction, and metal binding site prediction, which allow researchers to obtain a more comprehensive understanding of the presence and function of various metals in different organisms (24–27). However, due to the distinct utilization mechanisms between Se and metals, such deep learning-based strategies have not been applied for the identification of selenoprotein genes yet.

In this study, we adopted the idea of Bidirectional Encoder Representations from Transformers (BERT) model, which has recently made great success in many areas such as natural language processing (NLP) and biomedical text mining and developed a deep learning-based algorithm (called deep-Sep) for the prediction of bacterial selenoprotein genes in genomic databases. This predictor overcomes those drawbacks of previous methods and achieves much better performance than ever before. We used this tool to screen a selected number of bacterial genomes containing Sec machinery genes and further analyzed the selenogenomes of these organisms. Our approach provides a promising solution for precise and efficient identification of bacterial selenoprotein genes without relying on predefined SECIS elements or expensive calculations.

## RESULTS

### The overall performance of deep-Sep

**Fig 1:**
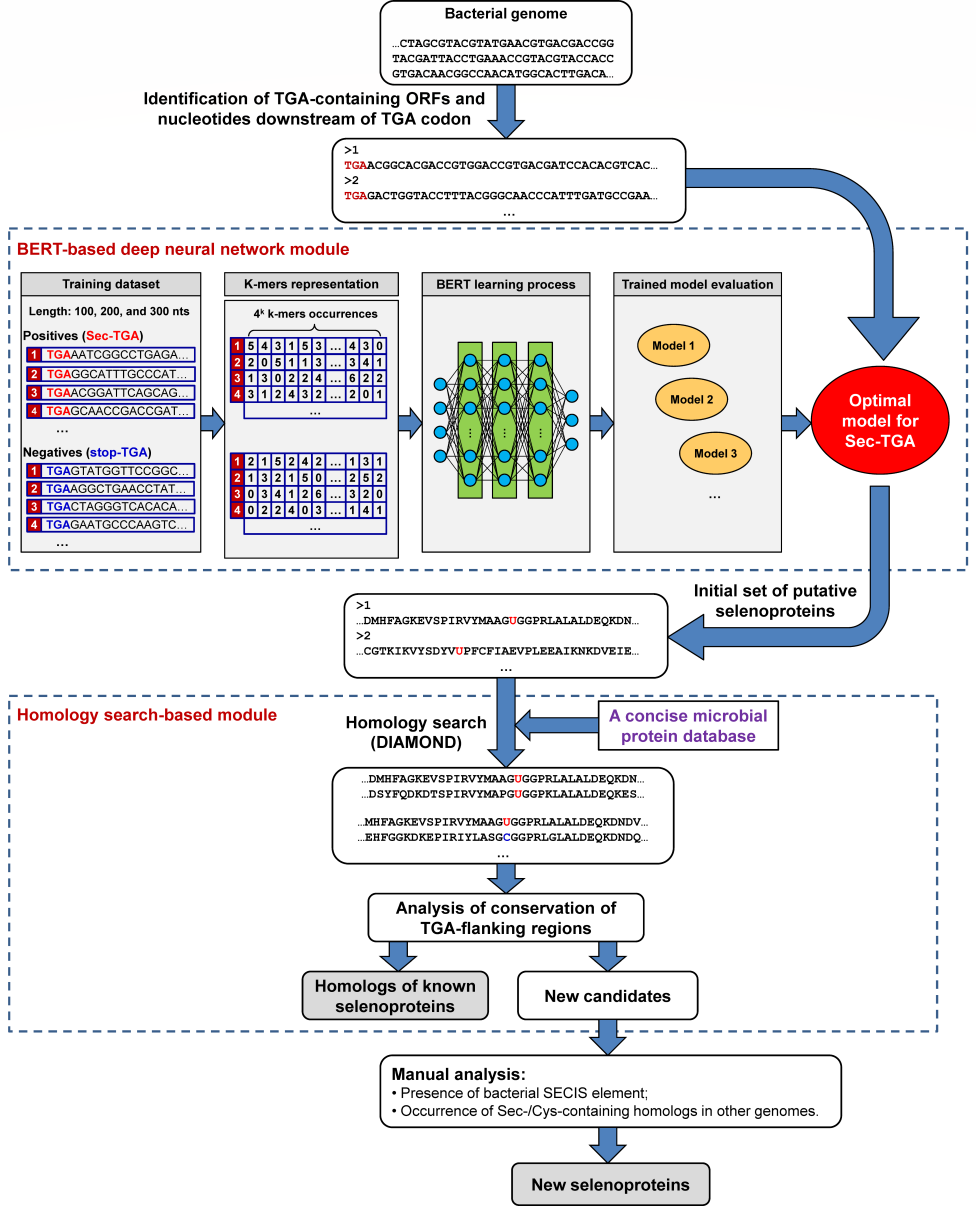
A schematic overview of the deep-Sep algorithm. The procedure consists of two modules: BERT-based deep neural network module and homology search-based module. Details of the search process are introduced in the text.

A general scheme of the deep-Sep algorithm is shown in Fig. 1. It consists of two parts: a BERT-based deep neural network module and a homology search-based module. In the first module, the neural network model for Sec-TGA prediction has been pre-trained based on the exploration of potential features associated with Sec-TGA decoding in sequences immediately downstream of TGA codon from a large number of known bacterial selenoprotein genes (positives) and the same number of non-selenoprotein genes (negatives). The initial set of predicted Sec-TGA codons and selenoprotein genes is then filtered by the second module to produce a more reliable set of selenoprotein genes.

To train and evaluate the performance of the deep learning-based Sec-TGA model, we tried three different sequence lengths (100, 200, and 300 nucleotides [nt]) to develop corresponding models during the pre-training phase. For each model, we employed a k-mer representation (with k values of 3, 4, 5) to generate feature matrices for both positive and negative data sets, which may guide the classifier design. After assessing all nine combinations of different sequence lengths and k-mer sizes (namely, 100 nt/3-mer, 100 nt/4-mer, 100 nt/5-mer, 200 nt/3-mer, 200 nt/4-mer, 200 nt/5-mer, 30 0nt/3-mer, 300 nt/4-mer, and 300 nt/5-mer) using the test set, we found that the 300 nt/3-mer model achieved the highest values for most metrics, including accuracy (0.940), recall (0.922), *F*_1_ score (0.939, a metric combining precision and recall to assess the model’s overall accuracy), and area under the receiver operating characteristic curve (AUROC, 0.987) (Table 1). Thus, it is considered the optimal model for Sec-TGA recognition and subsequently utilized for the analysis of selenogenomes within a collection of bacterial genomes.

**TABLE 1:** Comparison of the performance of BERT-based Sec-TGA models with different combinations of sequence lengths and k-mers on the test data set

Sequence length (nt)	k-mer value	Accuracy	Precision	Recall	*F*_1_ score	AUROC
100	3	0.931	0.957	0.904	0.929	0.985
	4	0.932	**0.965** ^*a*^	0.896	0.929	0.986
	5	0.919	0.939	0.896	0.917	0.978
200	3	0.920	0.953	0.883	0.917	0.983
	4	0.925	0.961	0.886	0.922	0.985
	5	0.916	0.952	0.876	0.912	0.979
300	3	**0.940**	0.956	**0.922**	**0.939**	**0.987**
	4	0.903	0.947	0.854	0.898	0.976
	5	0.862	0.938	0.775	0.849	0.951

^*a*^ The highest value for each metric is shown in bold.

Although our model utilizes a deep learning framework to automatically learn features instead of manually curated features used in the bSECISearch program for the identification of Sec-TGA codon and selenoprotein genes, a certain number of non-selenoprotein genes are incorrectly predicted as selenoprotein genes. This suggests that the current 300 nt/3-mer model may lack the necessary precision to completely eliminate false Sec-TGA codons and requires further refinement. Additionally, it is possible that there are unknown mechanisms beyond the 300 nts downstream of the Sec-TGA codon that differentiate it from stop-TGA codons. The homology search-based module is then used to remove these false positives. Although this module alone could potentially identify selenoprotein genes encoded in a bacterial genome, it would require significantly more computational time compared to our current algorithm, ranging from tens to even hundreds of times more. This is due to the need to use all valid open reading frames (ORFs, nucleotide triplet sequences that begin with a start codon (ATG, CTG, or GTG in bacteria) and terminate with a stop codon) containing potential in-frame TGA codons as queries for the homology search. We examined all non-selenoprotein genes that were misclassified as selenoprotein genes by our optimal Sec-TGA model and found that they could all be filtered out by the homology search-based module within minutes. Thus, the deep-Sep algorithm appears to be an efficient and reliable approach for the prediction of selenoprotein genes in bacteria.

### Identification of known selenoprotein genes in independent test Sec-utilizing bacteria

As our initial application of this approach, we analyzed sequenced genomes of 20 Sec-utilizing bacteria from different clades. These independent test organisms were pre-selected and have not been used for the training of the neural network model (see Materials and Methods). We first carried out an exhaustive homology search against these genomes using the tblastn program with previously reported prokaryotic selenoprotein families (Table S1). A total of 298 selenoprotein sequences belonging to 56 selenoprotein families were identified (Table 2). Among them, only 195 selenoprotein genes have been correctly annotated in their genomes released by NCBI.

**TABLE 2:** Analyses of sequenced genomes of 20 independent test Sec-utilizing bacteria with deep-Sep

Clade	Organism	GenBank accession number	Genome size (nt)	Number of TGA	All known selenoprotein genes	deep-Sep algorithm		Final results	
						Deep neural network module	Homology search-based module	Detected known selenoprotein genes	New selenoprotein genes
Acidobacteriota	*Occallatibacter riparius* DSM 25168	GCA_025264625.1	6,794,547	199,193	3	4,755	3	3	0
Alphaproteobacteria	*Afipia carboxidovorans* OM5	GCA_000218565.1	3,896,074	111,282	3	1,582	3	3	0
Candidate division LCP-89	*Candidate division LCP-89 bacterium* B3_LCP	GCA_005223185.1	3,794,239	150,290	4	2,291	4	4	0
Candidate division LCP-89	*Candidate division LCP-89 bacterium* M8-62_Bin_262	GCA_025774735.1	4,076,731	143,118	7	3,446	7	7	0
Spirochaetota	*Treponema pedis* str. T A4	GCA_000447675.1	2,889,325	73,154	9	568	9	9	0
Spirochaetota	*Treponema* sp. OMZ 790	GCA_024181285.1	3,093,697	94,737	8	798	8	8	0
Synergistota	*Aminobacterium* sp. MB27-C1	GCA_030908405.1	2,427,830	82,829	13	1,113	13	13	0
Synergistota	*Cloacibacillus porcorum* CL-84 (T)	GCA_001701045.1	3,585,187	106,647	18	1,893	18	18	0
Thermodesulfobacteriota/Desulfobacteria	*Desulfosarcina ovata* subsp. ovata oXyS1	GCA_009689005.1	7,630,248	261,817	24	4,435	24	24	0
Thermodesulfobacteriota/Desulfobacteria	*Desulfosarcina* sp. BuS5	GCA_028752835.1	4,175,088	159,262	15	1,702	18	15	3
Thermodesulfobacteriota/Desulfobacteria	*Desulfosarcina widdelii* PP31	GCA_009688965.1	7,297,718	230,838	38	4,090	38	37	1
Thermodesulfobacteriota/Desulfobulbia	*Desulfolithobacter dissulfuricans* GF1	GCA_025998535.1	3,596,428	116,503	22	2,524	22	22	0
Thermodesulfobacteriota/Desulfobulbia	*Desulfosediminicola flagellatus* IMCC35005	GCA_005116655.2	6,751,878	289,141	43	4,451	46	43	3
Thermodesulfobacteriota/Desulfobulbia	*Desulfosediminicola ganghwensis* IMCC35004	GCA_005116675.2	5,653,142	227,166	40	4,970	45	40	5
Thermodesulfobacteriota/Desulfovibrionia	*Desulfomicrobium orale* DSM 12838	GCA_001553625.1	2,783,374	79,615	6	1,367	6	6	0
Thermodesulfobacteriota/Desulfovibrionia	*Salidesulfovibrio onnuriiensis* IOR2	GCA_008001235.1	3,886,744	108,061	10	1,375	10	10	0
Thermodesulfobacteriota/Desulfuromonadia	*Desulfuromonas soudanensis* WTL	GCA_001278055.1	3,958,620	107,038	13	2,064	13	13	0
Thermodesulfobacteriota/Desulfuromonadia	*Geobacter sulfurreducens* PL	GCA_024205665.1	3,783,299	113,712	13	2,464	13	13	0
Thermodesulfobacteriota/Desulfuromonadia	*Syntrophotalea acetylenica* DSM 3246	GCA_001888165.1	3,206,049	93,125	5	1,693	5	5	0
Thermodesulfobiota	*Thermodesulfobium* sp. clean6954	GCA_027452805.1	1,561,321	55,221	4	570	4	4	0
Total			84,841,539	2,802,749	298	48,151	309	297	12

To predict selenoprotein genes, deep-Sep collects all TGA triplets in the six reading frames (on both strands) of each query genome and examines the regions upstream and downstream of each TGA triplet for the presence of a valid ORF. For those ORFs containing possible in-frame TGA codons, 300 nts immediately downstream of the TGA codon are retrieved and quickly scanned by the optimal Sec-TGA model to obtain an initial set of putative Sec-TGA codons and selenoprotein-encoding ORFs. In this study, more than 98% of total TGA triplets in each of the examined bacterial genomes were removed, resulting in the remaining 48,151 TGA triplets. Subsequent application of the homology search-based module resulted in 309 candidate selenoprotein genes. These hits were further divided into homologs of previously known selenoproteins (3 to 43 sequences in different organisms) and new candidates. Here, the average running time for screening a single bacterial genomic data set by deep-Sep is below 10 minutes. Except for one selenoprotein (named hypothetical protein OS_HP3) in *Desulfosarcina widdelii* PP31, all known selenoprotein genes in these examined bacterial genomes could be identified by deep-Sep. We also analyzed these organisms using the existing state-of-the-art method, bSECISearch. It took more than 24 hours for the screening of each bacterial genome, and at least 40 known selenoprotein genes (13.0%) in these genomes could not be identified by bSECISearch (Fig. S1). Therefore, it is evident that, compared to bSECISearch, the deep-Sep algorithm exhibits superior performance in terms of both speed and accuracy when predicting known selenoprotein genes.

It is worth noting that two *Desulfosediminicola* species were found to have ≥40 known selenoprotein genes (43 and 40 selenoprotein genes in *D. flagellatus* IMCC35005 and *D. ganghwensis* IMCC35004, respectively), which have exceeded the largest bacterial selenogenome previously observed in *Syntrophobacter fumaroxidans MPOB* (39 selenoprotein genes) (28). Thus, the record of the size of selenogenomes in bacteria has been renewed by this study.

### Prediction of new selenoproteins in bacteria

**Fig 2:**
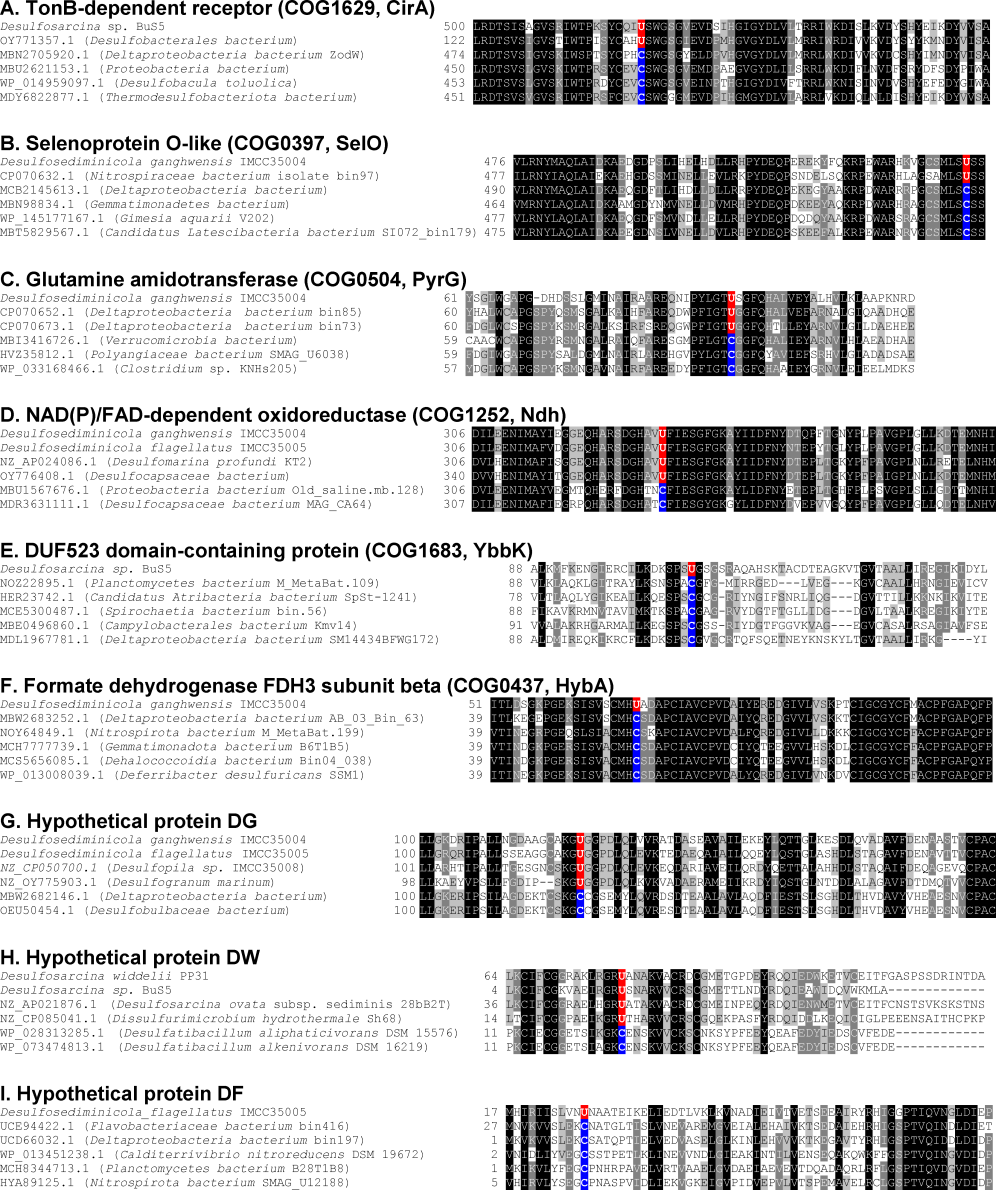
Multiple sequence alignments of new selenoproteins and their Cys-containing homologs. The alignments show Sec-flanking regions in new selenoprotein sequences predicted in examined bacterial genomes and their Sec-/Cys-containing homologs in other organisms. Predicted Sec (U) and the corresponding Cys (C) residues are shown in red and blue backgrounds, respectively. Other residues shown in white on black or grey are conserved in these proteins. (A) TonB-dependent receptor (COG1629, CirA); (B) selenoprotein O-like (COG0397, SelO); (C) glutamine amidotransferase (COG0504, PyrG); (D) NAD(P)/FAD-dependent oxidoreductase (COG1252, Ndh); (E) DUF523 domain-containing protein (COG1683, YbbK); (F) formate dehydrogenase FDH3 subunit beta (COG0437, HybA); (G) hypothetical protein DG; (H) hypothetical protein DW; (I) hypothetical protein DF.

**Fig 3:**
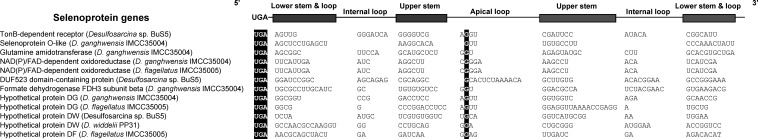
Alignment of SECIS elements present in new bacterial selenoprotein genes. The SECIS elements were predicted by using the bSECISearch program. Conserved nucleotides in the majority of known bacterial SECIS elements are highlighted in black.

Besides known selenoproteins, a total of 12 new selenoprotein genes (belonging to nine families) were also identified in some of these test Sec-utilizing bacteria by deep-Sep (Table 3, details can be found in Table S2). The majority of them contain conserved domains with known functions, such as TonB-dependent receptor (COG1629, CirA), glutamine amidotransferase (COG0504, PyrG), and NAD(P)/FAD-dependent oxidoreductase (COG1252, Ndh). In addition, three kinds of hypothetical proteins with no conserved domains were also detected and further named according to the organisms containing them (named hypothetical protein DG, hypothetical protein DW, and hypothetical protein DF). All of these new selenoproteins have a number of Cys-containing homologs in a variety of organisms. Moreover, except for DUF523 domain-containing protein, formate dehydrogenase FDH3 subunit beta, and hypothetical protein DF, all selenoproteins have TGA-containing homologs in some other Sec-utilizing organisms. Multiple sequence alignments of these new selenoproteins, along with their Sec-/Cys-containing homologs, demonstrate sequence conservation of Sec/Cys pairs and their flanking regions (Fig. 2). Further examination of bacterial SECIS elements for these selenoprotein genes revealed that they have bacterial SECIS-like structures immediately downstream of the putative Sec-TGA codons (Fig. 3). Interestingly, Sec-containing homologs of selenoprotein O (SelO), which were previously thought of as a eukaryotic selenoprotein family, were first detected in certain bacteria (such as *D. ganghwensis* IMCC35004). It seems that this selenoprotein family has also evolved in prokaryotes, probably from Cys-containing homologs. Among the 20 examined bacteria, the largest selenogenome was observed in *D. flagellatus* IMCC35005, which contains at least 46 selenoprotein genes. Further work is needed to verify these newly identified selenoprotein genes and to explore the selenogenomes of more Sec-utilizing bacteria using deep-Sep.

**TABLE 3:** New selenoproteins detected in bacteria

Protein name (domain)	Organism	Presence of TGA-containing homologs in other Sec-utilizing bacteria	Presence of Cys-containing homologs in bacteria
TonB-dependent receptor (COG1629, CirA)	*Desulfosarcina* sp. BuS5	Yes	Yes
Selenoprotein O-like (COG0397, SelO)	*Desulfosediminicola ganghwensis* IMCC35004	Yes	Yes
Glutamine amidotransferase (COG0504, PyrG)	*Desulfosediminicola ganghwensi*s IMCC35004	Yes	Yes
NAD(*P*)/FAD-dependent oxidoreductase (COG1252, Ndh)	*Desulfosediminicola ganghwensis* IMCC35004 *Desulfosediminicola flagellatus* IMCC35005	Yes	Yes
DUF523 domain-containing protein (COG1683, YbbK)	*Desulfosarcina* sp. BuS5	No	Yes
Formate dehydrogenase FDH3 subunit beta (COG0437, HybA)	*Desulfosediminicola ganghwensis* IMCC35004	No	Yes
Hypothetical protein DG	*Desulfosediminicola ganghwensis* IMCC35004 *Desulfosediminicola flagellatus* IMCC35005	Yes	Yes
Hypothetical protein DW	*Desulfosarcina* sp. BuS5 *Desulfosarcina widdelii* PP31	Yes	Yes
Hypothetical protein DF	*Desulfosediminicola flagellatus* IMCC35005	No	Yes

We also checked if our algorithm can distinguish the Sec-encoding function of the TGA codon from other coding functions. It has been known that TGA encodes tryptophan in bacteria of the *Mycoplasma* (now called *Mycoplasmoides*) genus (29). We analyzed the genomes of two *Mycoplasmoides* species, *M. genitalium* and *M. gallisepticum* (containing 25,382 and 43,498 TGA triplets, respectively). Although the BERT-based deep neural network module produced 265 (*M. genitalium*) and 419 (*M. gallisepticum*) Sec-TGA candidates, the subsequent homology search-based module discarded all of them. Thus, our method may also be used to distinguish the Sec-encoding function from other recoding functions of TGA codon.

### Development of a web-based interface for deep-Sep

A user-friendly web-based tool is provided that allows users to remotely predict selenoprotein genes in query nucleotide sequences within minutes. The web server interface is freely accessible to users at http://deepsep.metalbioinfolab.net:7001/.

## DISCUSSION

Selenoproteins are present in all three domains of life and are responsible for the majority of biological functions and molecular pathways of Se (17, 28, 30–32). So far, the number of selenoprotein families reported in bacteria is twice as large as that in eukaryotes. However, identification of selenoprotein genes in bacteria is still difficult compared to eukaryotes, mainly due to the lack of suitable models recapitulating various bacterial SECIS elements. Although the bSECISearch program, which is based on a common structural model for the majority of known bacterial SECIS elements, has been frequently used to predict new selenoprotein genes in bacterial genomes, one of the major deficiencies is the inability to detect atypical SECIS elements in a fraction of known selenoprotein genes, resulting in the incompleteness in characterization of selenogenomes in bacteria (13). Another weakness of the existing methods for predicting bacterial selenoprotein genes (including both SECIS-dependent and SECIS-independent approaches) is that it takes a long time to analyze a single bacterial genome, which might be unable to meet the requirements of rapidly growing genomic databases. In recent years, Transformer-based deep learning approaches have achieved superior performance in resolving various problems of biological sequence and structural analysis (33–35). In the present study, we propose a BERT-based neural network model for identifying Sec-TGA codons in bacteria. We then use this model to develop the deep-Sep algorithm, which combines deep neural network and homology search-based techniques to automatically detect selenoprotein genes in bacterial genomic data sets.

Our algorithm has been designed for routine investigations of bacterial genomes for the identification of selenoprotein genes. The deep neural network module introduces a paradigm of pre-training and fine-tuning to generate the optimal model for Sec-TGA. However, the use of this model alone appears not to be sufficient to precisely identify selenoprotein genes in bacterial genomic sequences because of the presence of a certain number of false positives. We found that the number of potential Sec-TGA codons predicted by our BERT-based model was positively correlated with genome size but not with the GC content of the examined bacterial genomes (Fig. S2), which is different from bSECISearch as a positive correlation was observed between the number of predicted bacterial SECIS-like structures and the GC content of query genomes (13). This implies that the intrinsic characteristics utilized by our model are different from bSECISearch, which is based on a predefined bacterial SECIS structural model. Considering that distinct classes of SECIS elements may exist in bacteria, our SECIS-free model demonstrates a greater tolerance for variations within the SECIS region and thus becomes a better choice for the recognition of selenoprotein genes across a wide variety of bacterial organisms. Further computational methods may be used to enhance the accuracy of our model.

To remove most false positives derived from the optimal Sec-TGA model, we adopt a homology search-based strategy which is similar to but much faster than bSECISearch (13). Our results are not only consistent with the exhaustive search results by using all known selenoprotein sequences, but also provide a substantial improvement in computational speed, reducing the running time from more than 24 hours to just a few minutes.

An important novelty of our study is the identification of nine new selenoprotein families in several bacterial genomes used as independent test organisms for selenoprotein gene prediction. Most of them have conserved functional domains, suggesting that Se might be involved in biological processes related to these functions. Sec-containing homolog of SelO, a eukaryotic selenoprotein family, was found in certain bacteria. It was predicted to be a pseudokinase due to the absence of the catalytic aspartate which is necessary for the phosphotransfer (36). Recent studies have shown that SelO catalyzes the covalent addition of AMP from ATP to the hydroxyl side chain of protein substrates in a post-translational modification known as AMPylation (37, 38). The identification of Sec-containing SelO-like proteins in bacteria suggests that the relationship between Se and AMPylation might be also present in this kingdom. On the other hand, although no conserved domains could be detected for three hypothetical protein families, they have passed the stringent criteria employed by deep-Sep and should be considered as promising new selenoprotein candidates. Therefore, our method can greatly help improve the understanding of the important roles of Se in bacteria.

It should be noted that currently the deep-Sep algorithm may not correctly identify selenoproteins that lack Cys-containing homologs. Even if such selenoprotein genes can be initially identified by the BERT-based neural network module, they may be filtered out by the homology search-based module. Previously, only one such protein, glycine reductase complex selenoprotein A, was thought to lack Cys-containing homologs (39). However, in this study, we detected several Cys-containing homologs for this selenoprotein (Fig. S3). Now it seems that all known selenoproteins have Cys-containing homologs. Thus, our algorithm represents a fast, efficient, and reliable method for the identification of selenogenomes in various bacterial genomes. In the future, more accurate models for Sec-TGA codons and selenoprotein genes should be developed to improve the accuracy of selenoprotein gene prediction.

In conclusion, we have developed a Transformer-based deep learning model for Sec-TGA codon recognition in bacteria and subsequently built an automated tool for predicting selenoprotein genes in bacterial genomic data sets. Our method has the potential to accurately annotate selenoprotein genes in various bacterial genomic and metagenomic sequencing projects. Systematic characterization of selenoprotein genes and selenogenomes may provide a better understanding of the biological functions of Se in nature.

## MATERIALS AND METHODS

### Genomic sequences and resources

Completely and nearly completely sequenced genomes and corresponding protein-coding genes from organisms belonging to diverse bacterial clades were downloaded from the NCBI GenBank database. A total of 278,451 organisms (including various species and strains) were collected (as of December 2022).

Blast programs (version 2.14.0) were downloaded from the NCBI ftp server (ftp://ftp.ncbi.nih.gov/blast/). Multiple sequence alignment was performed using the ClustalW program in MEGA (version 11) (40), and then edited with the GeneDoc program (version 2.7, available at https://github.com/karlnicholas/GeneDoc).

### Identification of Sec-utilizing organisms and known selenoprotein genes in bacteria

We used *Escherichia coli* SelA, SelB, and SelD sequences (Table S1) as queries to search for components of the Sec encoding system in bacteria. The tblastn program was used to search against the genomic sequences for homologs with e-value <0.001. Iterations of tblastn searches were then performed within each clade (phylum or class) to identify additional homologous sequences using selected sequences from the same or closely related clades in the primary data set. Moreover, the iterative profile hidden Markov model (profile-HMM) search tool, JackHMMER, was also used to search for distant homologs that match the HMMs of query proteins with e-value <0.05 (41). All obtained sequences were analyzed with the rpsblast program to verify orthologous genes by examining their conserved domain information (such as COG and Pfam) (42, 43). Considering that SelD is a selenoprotein in many organisms, the presence of Sec-containing SelD in bacteria was also verified by using the strategy for the identification of selenoprotein genes (see below). As SelB has been suggested as the signature of Sec utilization trait (44), organisms that use Sec were further verified by the requirement for (i) the presence of SelB, or (ii) the presence of SelA and SelD genes. A total of 83,103 Sec-utilizing organisms were identified. We randomly excluded a small number of clades (including *Thermosulfidibacterota*, *Thermodesulfobiota*, and candidate division LCP-89) from the model development process. Instead, they were reserved for model assessment, allowing for a potentially more impartial evaluation of the general applicability of our model across diverse taxonomic groups. Additionally, we manually selected 20 organisms from various bacterial taxa to serve as independent test organisms for the prediction of all selenoprotein genes in their genomes. These organisms mainly included those belonging to previously reported selenoprotein-rich clades like *Synergistota* and *Thermodesulfobacteriota* (28), as well as organisms from the aforementioned clades that were not utilized for model development. The remaining organisms were used for model training and evaluation.

We also collected representative sequences for previously reported prokaryotic selenoprotein families (Table S1) (14, 16, 17, 45, 46). These sequences were used to search against Sec-utilizing bacterial genomes for selenoprotein homologs via tblastn with a cutoff e-value of 0.001. Distant homologs were further identified by using additional iterations of tblastn searches in different clades. The tblastn output for each selenoprotein sequence was parsed, and only the Sec/TGA pairs (Sec in the query sequence was aligned with TGA in the nucleotide sequence from the target genomic data set) were chosen. A valid ORF was predicted for each in-frame Sec-TGA codon. Redundant selenoprotein sequences were removed.

### Data preprocessing and data set preparation

The nucleotide sequence downstream of the Sec-TGA codon is known to be critical for recoding of TGA as Sec, which is suitable for the construction of a deep learning-based model for Sec-TGA recognition; however, the appropriate sequence length needs to be explored. To address this, we extracted 100, 200, and 300 nts immediately downstream of the Sec-TGA codon in each of the known selenoprotein genes identified above. The obtained sequence data set was examined to eliminate redundant sequences and subsequently split into several positive subsets, including (i) a training set containing 90% of sequences from 40 randomly selected selenoprotein families (number of sequences: 100 nt: 51,818; 200 nt: 56,037; 300 nt: 58,701); (ii) a validation set consisting of 50% of sequences from 30 randomly selected selenoprotein families out of the remaining pool (number of sequences: 100 nt: 16,921; 200 nt: 19,771; 300 nt: 21,701); (iii) a test set comprising unselected sequences from the aforementioned 70 selenoprotein families as well as sequences from other unused selenoprotein families, after excluding sequences with a sequence identity of ≥70% to those included in the training and validation sets (number of sequences: 100 nt: 809; 200 nt: 784; 300 nt: 1,064). The test set also encompassed organisms belonging to clades that were excluded from the training and validation phases of our model (with the exception of those organisms that were pre-selected as part of the independent test organisms).

With regard to negative data sets, we first randomly selected 6,000 bacteria that do not use Sec and collected all annotated genes that end with TGA (stop-TGA codon). Similar to the preprocessing for selenoprotein genes, we extracted 100, 200, and 300 nts downstream of the stop-TGA codon in their genomes and produced a preliminary negative data set. In order to include diverse gene families, we used the easy-linclust module in the MMseqs2 software (version 15–6f452) to cluster all sequences at a threshold of 50% sequence identity and 80% coverage (47). The remaining sequences were randomly assigned to generate negative data sets for the training, validation, and test sets, respectively, which contained the same number of sequences as the corresponding positive set.

### Composition of deep-Sep

As shown in Fig. 1, the deep-Sep algorithm is composed of two modules: (i) a pre-trained BERT-based deep neural network module, which is responsible for initial identification of Sec-TGA codons and bacterial selenoprotein genes in genomic sequences, and (ii) a homology search-based module which filters out false positives by sequence homology searches.

#### BERT-based deep neural network module

We tokenized sequences in both positive and negative data sets with the k-mer (k = 3, 4, 5) representation, which has been widely used in analyzing DNA sequences, to make a training input for a BERT-based deep learning architecture (48).

BERT is a Transformer-based technique for pre-training contextual word representations that enables state-of-the-art results across a wide range of NLP tasks (49, 50). It includes two separate stages, pre-training and fine-tuning, which may develop general understandings from massive amounts of unlabeled data and then solve various applications with minimal task-specific architectural changes.

In the pre-training phase, this BERT-based neural network module first takes a set of unlabeled nucleotide sequences tokenized with k-mer representation as input and captures contextual information by performing the self-attention mechanism. We used a batch size of 32 and pre-trained for three epochs over the label-free training data. The tokens were projected into an embedding vector with 768 dimensions and fed to six subsequent self-attention encoders. Each encoder contained a multi-head self-attention layer with 768 hidden units and six attention heads. In addition, the intermediate layers of the encoder were equipped with 2048 hidden units. In order to reduce the risk of overfitting, a dropout rate of 0.2 was applied over all the hidden and attention layers. Lastly, a fully connected layer followed by a Softmax activation function was used as the output layer of the model for classification.

In the fine-tuning phase, we started from the pre-trained parameters and fine-tuned our model using the same labeled training data set. The model was trained for 10 epochs using the cross-entropy loss function and the AdamW optimizer with a batch size of 128 and a learning rate of 1e-6. After each training epoch, we assessed the model over the validation set to monitor the convergence. The model with the highest accuracy on the validation set was retained for subsequent testing. The performance of the predictive model was evaluated using accuracy, precision, recall, *F*_1_ score, and AUROC for the test set.

To ascertain the optimal model from different lengths of nucleotide sequence downstream of Sec-TGA and their k-mer representations, a total of nine length- and k-mer-based models with the same architectures and hyperparameters were compared. The model with the highest evaluation metrics on the test set was chosen as the final model for predicting Sec-TGA codons.

#### Homology search-based module

This module makes use of homology search strategy to identify Sec-/Cys-containing homologs of putative selenoproteins in microbial genomes, which may help to remove most false positives from the primary set of predicted selenoprotein genes (13). The key process of the procedure is to examine the conservation of Sec-TGA-flanking regions in each putative selenoprotein sequence.

Considering that the standard NCBI non-redundant protein database has grown rapidly and is now very large (over 300 million sequences), it is better to build a concise protein sequence database with a set of representative microbial organisms to improve the efficiency of homology search. Based on bacterial genomic data downloaded from NCBI, we randomly selected 1– 5 organisms for a wide range of bacterial clades (classes or orders) according to the number of sequenced organisms in each clade, resulting in the collection of 3,326,489 protein sequences derived from 871 organisms. In addition, we selected 1–5 representative selenoprotein sequences for each known prokaryotic selenoprotein family and included them in the data set. The final database consists of a total of 3,326,820 protein sequences.

The DIAMOND program (version 2.1.8), a fast and sensitive protein aligner, is used to screen the concise microbial protein database with each of the predicted selenoprotein sequences (51). Only hits with a cutoff e-value of 0.01 and a sequence identity of at least 20% are selected. These hits are then analyzed to assess the conservation of TGA-flanking regions and residues aligned with predicted Sec in the query sequence. The selenoprotein-encoding ORFs are retained if the following criteria could be satisfied: (i) the presence of at least one known Sec-containing homolog or (ii) at least two of the top ten hits are Cys-containing homologs in different organisms. The resulting selenoprotein candidate set can be divided into homologs of known selenoproteins (including experimentally validated and theoretically predicted selenoproteins in the literature or the GenBank database) and new candidates. All new selenoprotein candidates are manually analyzed for the presence of bacterial SECIS elements downstream of the TGA codon and the occurrence of Sec-/Cys-containing homologs in other bacterial genomes. Finally, a set of new selenoprotein genes could be generated.

### Implementation of deep-Sep

The BERT-based deep neural network module of deep-Sep is written in Python (version 3.9.7) based on the PyTorch framework and the transformers package (version 4.34.1). We trained our models on one machine equipped with NVIDIA Tesla A100 GPU and Linux platform. The homology search-based module of deep-Sep and other scripts used in this study are also written in Python using different packages. The deep-Sep program is completely automated and has been successfully tested on the Linux platform.

## Data Availability

All known selenoprotein sequences detected in the 20 test organisms are available in Data S1. The concise microbial protein database for DIAMOND search is available at https://drive.google.com/file/d/1G1wMizJbmnU-vfqIbVjNcH7yyEYmbXVl/view?usp=drive_link. The deep-Sep algorithm is implemented in Python using PyTorch, and all custom source codes used in this study are available at https://github.com/ZhangBioLab/deepSep.

## SUPPLEMENTAL MATERIAL

Data S1 msystems.01258-24-s0001.txt All known selenoprotein sequences detected in the 20 test organisms.

Supplemental figures msystems.01258-24-s0002.pdf Figures S1 to S3.

Table S1 msystems.01258-24-s0003.xlsx Representative sequences for the bacterial Sec encoding system and previously reported selenoprotein families in prokaryotes.

Table S2 msystems.01258-24-s0004.xlsx New selenoproteins predicted in the test Sec-utilizing bacteria examined in this study.

ASM does not own the copyrights to Supplemental Material that may be linked to, or accessed through, an article. The authors have granted ASM a non-exclusive, world-wide license to publish the Supplemental Material files. Please contact the corresponding author directly for reuse.
