# Supplementary material for: deep-Sep: a deep learning-based method for fast and accurate prediction of selenoprotein genes in bacteria
Source: mSystems. 2025 Mar 10;10(4):e01258-24. doi: 10.1128/msystems.01258-24 (PMC12013277; doi:10.1128/msystems.01258-24)
Supplement: Supplemental figures — Figures S1 to S3. [file msystems.01258-24-s0002.pdf]

Supplementary figures

Fig. S1. Comparison of the performance on predicting known selenoprotein genes in the 20 test bacterial genomes between deep-Sep and bSECISearch. (A) Average running time; (B) Fraction of true positives.

Fig. S2. Scatter plots displaying the correlation between the number of Sec-TGA codons predicted by BERT-based model and some genomic characteristics of organisms examined here. (A) Genome size; (B) GC content. Correlation coefficients (CCs) are shown.

Fig. S3. Multiple alignment of glycine reductase complex selenoprotein A. Predicted Sec (U) and the corresponding Cys (C) residues are shown in red and blue backgrounds, respectively. Other residues shown in white on black or grey are conserved in these proteins.

Fig. S1

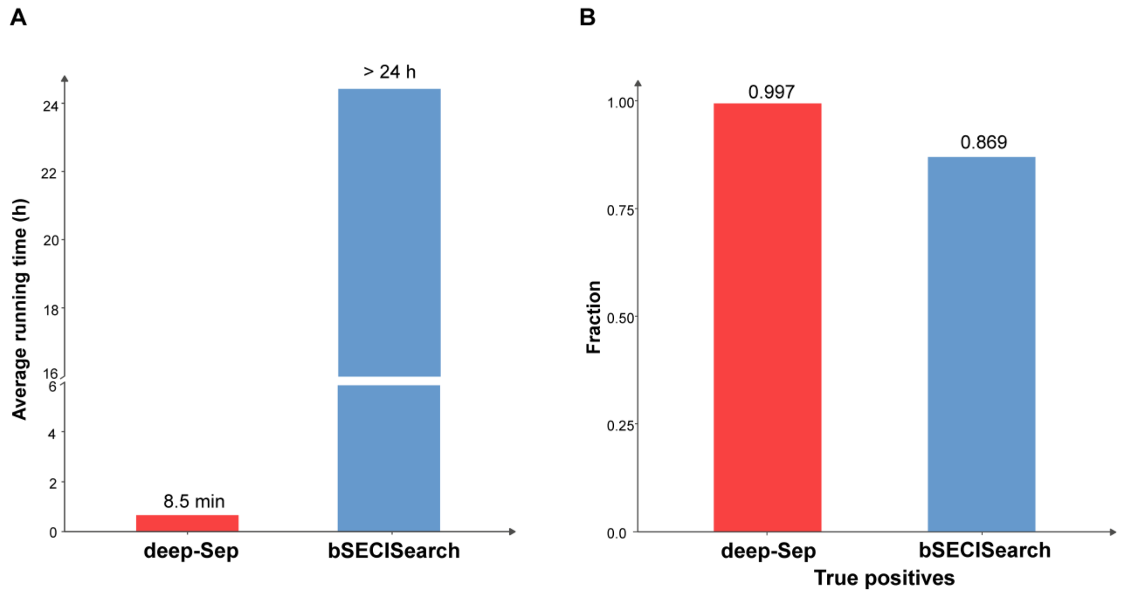
A
B
> 24 h
8.5 min
Average running time (h)
24
22
20
18
16
6
4
2
0
deep-Sep
bSECISearch
0.997
0.869
Fraction
1.00
0.75
0.50
0.25
0.0
deep-Sep
bSECISearch
True positives

Fig. S2

A

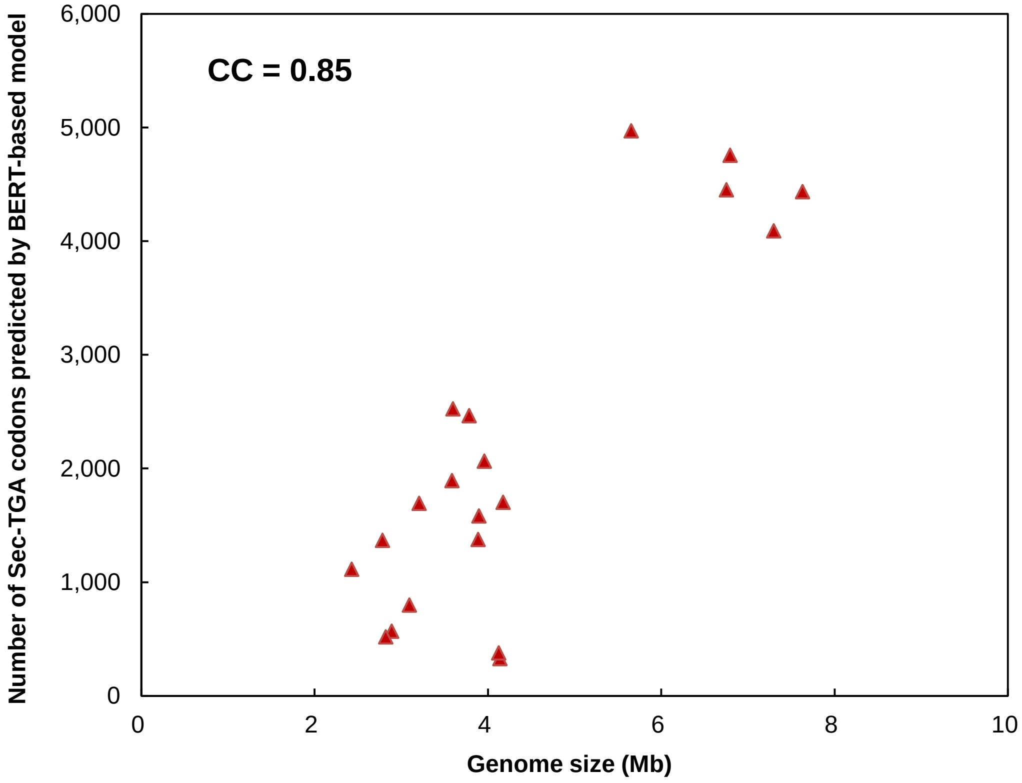

CC = 0.85
Number of Sec-TGA codons predicted by BERT-based model
Genome size (Mb)
6,000
5,000
4,000
3,000
2,000
1,000
0
0
2
4
6
8
10

B

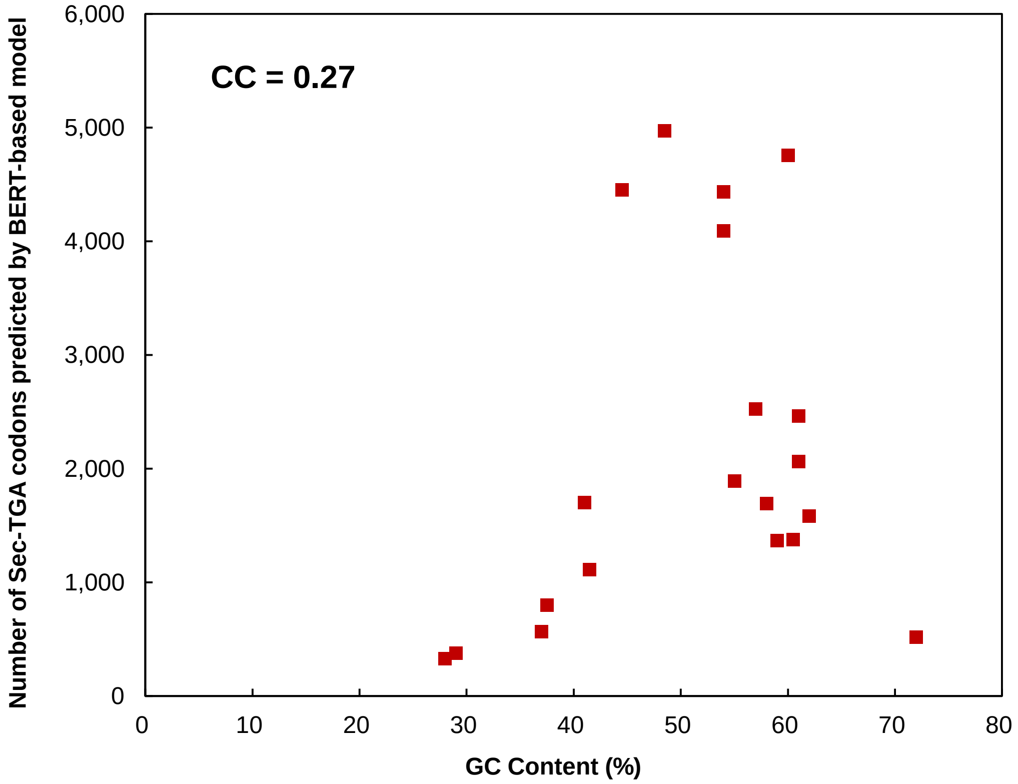

CC = 0.27
Number of Sec-TGA codons predicted by BERT-based model
GC Content (%)
6,000
5,000
4,000
3,000
2,000
1,000
0
0
10
20
30
40
50
60
70
80

Fig. S3

```
Desulfovibrio sp. 86                     1 -----METTARKIIVIGAMDGVPSEAISAALTESGHTVVFAINQFFVUMMAGAMDLEDQWKLVTVVKQFGNENLHVLLGCPDAES
Brevibacillus massiliensis               1 -----MVLEKKKVFVIGERDGVPAPAVAACVKASGADVIFMDTQCFVUTAAGAMDLEVQGALLKAVEQYGKENVVVMLGSPDADS
Desulfofundulus thermobenzoicus          1 ------MLKGKKVICIGERDGIPAPAIAECMRTAGAEPVLTLTQCFVUTAAGAMDLEDQGTIKKAVDEHGTENVVVVLGSPDAES
Anoxynatronum sibiricum                  1 ----MSFLDGKKVIIIGDRDGIPAPAMVECLKDTGAEVVFSSTECFVUTAAGAMDLENQNRVKTLTEKYGAENMLILLGAAEAES
Effusibacillus pohliae                   1 ------MLKGKKVFVVGERDGVPAPAVAECVTAAGGEVVFMDTQCFVUTAAGAMDLQVQEALKQAVEQYGRENVVVLLGSPDADS
Clostridium fermenticellae               1 ----MFMLKGKKVIAIGDRDGIPGPAIEACVKSAGAEVIFASTECFVUTAAGAMDLEIQQRVKDLTEKYGAENIVVILGGAEAEA
Treponema medium                         1 -----MELKGKKVIIIGDRDGIPGEAIKLCAETAGAEVVYAATECFVUTSAGAMDLENQKRVKDLAEKYGCENTVVLLGGAEAES
Candidatus Bathyarchaeota archaeon BA1   1 -----MKLGEKKIIIIGDRDGVHGEEIEDALKRMGYKPVFSCTECFVCTAAGSVDFPNQQKIKELAQTGRPEDFAVLLGVADSEG
Clostridium psychrophilum                1 ------MLKGKKVIVIGDKDGIPGASIEACVKSAKAEVVFSITKCFACSVVGAMDIELQQKVKDIALEYGAQNLVVVIGGSEAET
Clostridium sp.                          1 MIDPKKILAEKKVIIIGDKDGISGPAIEACLKSTGVEVVYVTTKCFACSTTGAMDVEIQQIIVDCATLHGAQNLVVVIGGSEAES
Clostridiaceae bacterium                 1 ------MLKGKKVIIIGDKDGIPAPTIGACLKTVGVEVVFSVTTCFTCSLAGAMDIENQQRIKDLASQYGEGNLAVILGGGDVET
Clostridium estertheticum                1 ----MKMFKSKKVIIIGDRDGIPGPAIEACVKSAKAQVVFSTTKCFSCSLAGAMDIELQQVVKDLTSKFGAENLVVVIGGAEAET
Clostridium frigoris                     1 ----MKMLKARKVIIIGDRDGIPGPAIEACVKSADAEVVFSTTKCFSCSLAGAMDNELQQIVKDLTAKHGAQNLVVIIGGAEAET

Desulfovibrio sp. 86                     81 SQIQGDTVSSGDPSLSGPLTDSQFYLQVHHVSEPEVRGFFSKEAFARHIEPFLSLVD---AEAIRQRMHELRGQMVQSKPRPMHG
Brevibacillus massiliensis               81 ADIYAETVTVGDPTYAGPLAGVELKLSVYHILEDEIKALIPEDVYQEQVGLMELSLD---KDAICEAMNNARKNFLG--------
Desulfofundulus thermobenzoicus          80 AEIYAETVTTGDPTFAGPLAGVSLGLPVYHILEDEIKQLVDPETYQQQVGMMEFVLD---KEAIVTSMKKIRQQSR---------
Anoxynatronum sibiricum                  82 AGLAAETVTNGDPTFAGPLAGVQLGLKVYHAVEPEFKDAVNPDVYDEQIGMMEMVLD---VDGIVEEMNNIRSEFCKY-------
Effusibacillus pohliae                   80 ADIYAETVTLGDPTYAGPLAGVSLRLAVYHILEDEVKQLVPEDVYQEQVGLAELSLD---KEAICEAMQNARARMSD--------
Clostridium fermenticellae               82 SGLSAETVSAGDPTFAGPLAGIELGLSVYHMVEPEVKQECDDAVYDEQCGMMEMVLD---VDEIISEVKTVRDEYSKY-------
Treponema medium                         81 SGLACETVTNGDPTFAGPLAGVSLGLLCYHVVEPEIKNQIDPKVYEEQISMMEMVLD---VPTIIAEVKEYREKYCKFLK-----
Candidatus Bathyarchaeota archaeon BA1   81 AEVHARTVTTGDPSYSGVLSGVELHLPVYHMFEPEVKNQVDKSVYDETIGVVEQSLNKKIVDDTIATVRRIREEGSAK-------
Clostridium psychrophilum                80 SGLCAETMANGDPTFVGPLSGIALGLTVYHVVEPEIKDECSETVYEKQCAVMEMVLE---IDEIILEVKSVRDKYSIY-------
Clostridium sp.                          86 SGLCAETVTTGDPTFVGPLAGIALGLVVYHVFEPEIKNECQESVYEEQCGIMEMVLE---IDEIILEVKSIRDKFSMYGLKRC--
Clostridiaceae bacterium                 80 CSITAETISAGDITEVGPLAGVSLGIPVYHIFEPEIRNECDLRVYEERCAIMEMVLD---VDKIVNEVRHIRLQYAQI-------
Clostridium estertheticum                82 SGITAETIAAGDPTFVGPLAGIALGLPVYHIFEPEIKEAFIKSVYDEQCSVMEMILD---IDEIIIEVKSFRDKFCKVSLKQ---
Clostridium frigoris                     82 SEVTAETIVAGDPTFVGPLAGIALGLAVYHVVEPEIKDSFVKSVYDEKCSIMEMVLD---IDEIIIEVKSLRDKFCKFSLK----
```
